# Robots facilitate human language production

**DOI:** 10.1038/s41598-021-95645-9

**Published:** 2021-08-18

**Authors:** Olga A. Wudarczyk, Murat Kirtay, Doris Pischedda, Verena V. Hafner, John-Dylan Haynes, Anna K. Kuhlen, Rasha Abdel Rahman

**Affiliations:** 1 Department of Psychology, Neurocognitive Psychology, Humboldt-Universität zu Berlin, Berlin, Germany; 2 Department of Computer Science, Adaptive Systems Group, Humboldt-Universität zu Berlin, Berlin, Germany; 3 Charité – Universitätsmedizin Berlin, corporate member of Freie Universität Berlin and Humboldt-Universität zu Berlin, Bernstein Center for Computational Neuroscience , Berlin, Germany; 4 Charité – Universitätsmedizin Berlin, corporate member of Freie Universität Berlin and Humboldt-Universität zu Berlin, Berlin Center for Advanced Neuroimaging , Berlin, Germany; 5 Faculty of Philosophy, Berlin School of Mind and Brain, Humboldt-Universität zu Berlin, Berlin, Germany

**Keywords:** Psychology, Computer science

## Abstract

Despite recent developments in integrating autonomous and human-like robots into many aspects of everyday life, social interactions with robots are still a challenge. Here, we focus on a central tool for social interaction: verbal communication. We assess the extent to which humans co-represent (simulate and predict) a robot’s verbal actions. During a joint picture naming task, participants took turns in naming objects together with a social robot (Pepper, Softbank Robotics). Previous findings using this task with human partners revealed internal simulations on behalf of the partner down to the level of selecting words from the mental lexicon, reflected in partner-elicited inhibitory effects on subsequent naming. Here, with the robot, the partner-elicited inhibitory effects were not observed. Instead, naming was facilitated, as revealed by faster naming of word categories co-named with the robot. This facilitation suggests that robots, unlike humans, are not simulated down to the level of lexical selection. Instead, a robot’s speaking appears to be simulated at the initial level of language production where the meaning of the verbal message is generated, resulting in facilitated language production due to conceptual priming. We conclude that robots facilitate core conceptualization processes when humans transform thoughts to language during speaking.

## Introduction

Recent developments in artificial intelligence have introduced autonomous and human-like robots into numerous aspects of everyday life. Natural social interactions with robots are however still far from expectations, emphasizing the need to advance human–robot social interaction as one of the currently most pressing challenges of the field of robotics^1^. In this study we focus on the increasingly more prevalent domain of interaction with robots: verbal communication^2,3^. We assess the extent to which a social robot’s verbal actions, in social interaction with humans, are simulated and predicted, or in other words *co-represented,* and explore the consequences of robot verbal co-representation on human language production. We focus on a social humanoid robot (Pepper, Softbank Robotics). Social robots, as physical agents, in contrast to other robots (e.g. service robots), have been developed specifically for interaction with humans^4^.

### Co-representation of human and robot task partners

In humans, co-representation is a central mechanism underlying human social interaction (e.g., Ref.^5^). Co-representation refers to the ability to represent the partner’s action alongside one’s own actions^6^. This supports the understanding of the partner’s intentions and enables the achievement of shared task goals. In social settings, humans seem to automatically co-represent the actions of their interaction partners (for reviews see, e.g., Refs.^7–9^). By observing a task partner’s action, corresponding motor representations automatically activate in the observer, allowing predictions of the partner’s behaviour and supporting coordination of the partner’s action with one’s own action^9^.

To achieve smooth human–robot interactions, humans and robots should be able to mutually predict and simulate each other’s behaviour^10^. Despite early findings suggesting that humans treat social technologies similarly to real people^11^, the intricacies of robot co-representation present a more complex picture (for a review see Ref.^10^). Early investigations on this topic predominantly focused on motor co-representation of non-human agents and suggested that the shared representational system is predominantly tuned to other humans^12,13^. Yet subsequent studies, exploring the additional role of beliefs about the interaction partner, revealed that motor co-representation of non-biological agents emerges if participants attribute *intentionality* to the agent*,* that is, the belief that the agent is acting in an active and intentional way^14–17^. Investigations in which participants completed real-life joint tasks with *specifically* humanoid robots corroborated these conclusions revealing that humans can co-represent a humanoid robot in a joint task (Social Simon Task^18^)^19,20^.

Co-representation, however, is not limited to lower levels of action control such as movements but extends to higher cognitive levels such as verbal communication. For instance, while our partner speaks, we covertly simulate and predict our partner's utterances^21^ which in turn, affects our own verbal behavior^22–26^. The understanding of the cognitive underpinnings of a social robot’s verbal co-representation is particularly important for the successful introduction of social robots across the range of verbal contexts, including daily interactions with social robots via speech (e.g., Ref.^27^), clinical contexts (e.g., Ref.^28^), learning environments (e.g., Ref.^29^) and social robots as social companions (e.g., Ref.^30^). Previous studies have not yet directly assessed the question of a social robot’s verbal co-representation in a joint naming task setting. However, studies on interactive situations with artificial agents provide important insights into language processing and language prediction. Firstly, while it was shown that humans communicate differently when they believe that their interaction partner is a computer (and not another human^31^), such differences seem to vanish with additional social cues^31^, and when interacting with human-like avatars in virtual reality settings (see Ref.^32^, for a review). Further, in virtual reality settings, sentence processing^33^ as well as the prediction of upcoming words were comparable in interactions with another human and human-like virtual agent^34,35^. With regards to humanoid robots, it was shown that humans adapt to their robot interaction partners by choosing words for everyday objects that match those of their artificial partner, a phenomenon referred to as lexical entrainment, reflecting a shift in language representations due to partner’s verbal behaviour^36–38^. This shift in language representation was found to persist even after the human–robot interaction ended (Ref.^38^, also note other forms of linguistic alignment for other types of artificial agents, e.g. Refs.^39–42^). At the same time, other studies report differences between human and social robot (specifically, the NAO robot) interaction partners with regards to peer-pressuring speakers to commit morphologically primed grammatical mistakes (i.e. morphological alignment^43^).

These lines of research provide the first evidence that a robot interaction partner can have an influence on a human's verbal behavior. What is not yet clear, however, is the extent to which such adaptations reflect a simulation of the partner’s verbal behavior via co-representation, as well as the nature of such a robot co-representation. Hence, in the current study we specifically address the question of whether and how a social robot’s verbal behaviour is simulated and thus co-represented in a joint language production task setting, and we explore the consequences of a robot’s verbal co-representation on human’s language production.

### Transforming thoughts to spoken words: the mechanisms of human language production

To assess the question of robot verbal co-representation, in the current study we build on theoretical and empirical insights from psycholinguistic research on speaking. In the following paragraphs, we describe how preverbal thoughts are transformed into articulated speech that makes our thoughts accessible to others, highlighting the core mechanisms of language production targeted in the current study.

Speaking begins with thinking—the conceptualisation of the speaker’s communicative intention with its meaning facets and associations^44–48^. The concepts and meaning facets are mapped onto corresponding words stored in the mental lexicon, from which co-activated words that best express the meaning of the intended message are selected. This process of meaning-based lexical selection is at the core of any act of speech production. Subsequently, the message is phonologically encoded and articulated.

**Figure 1:**
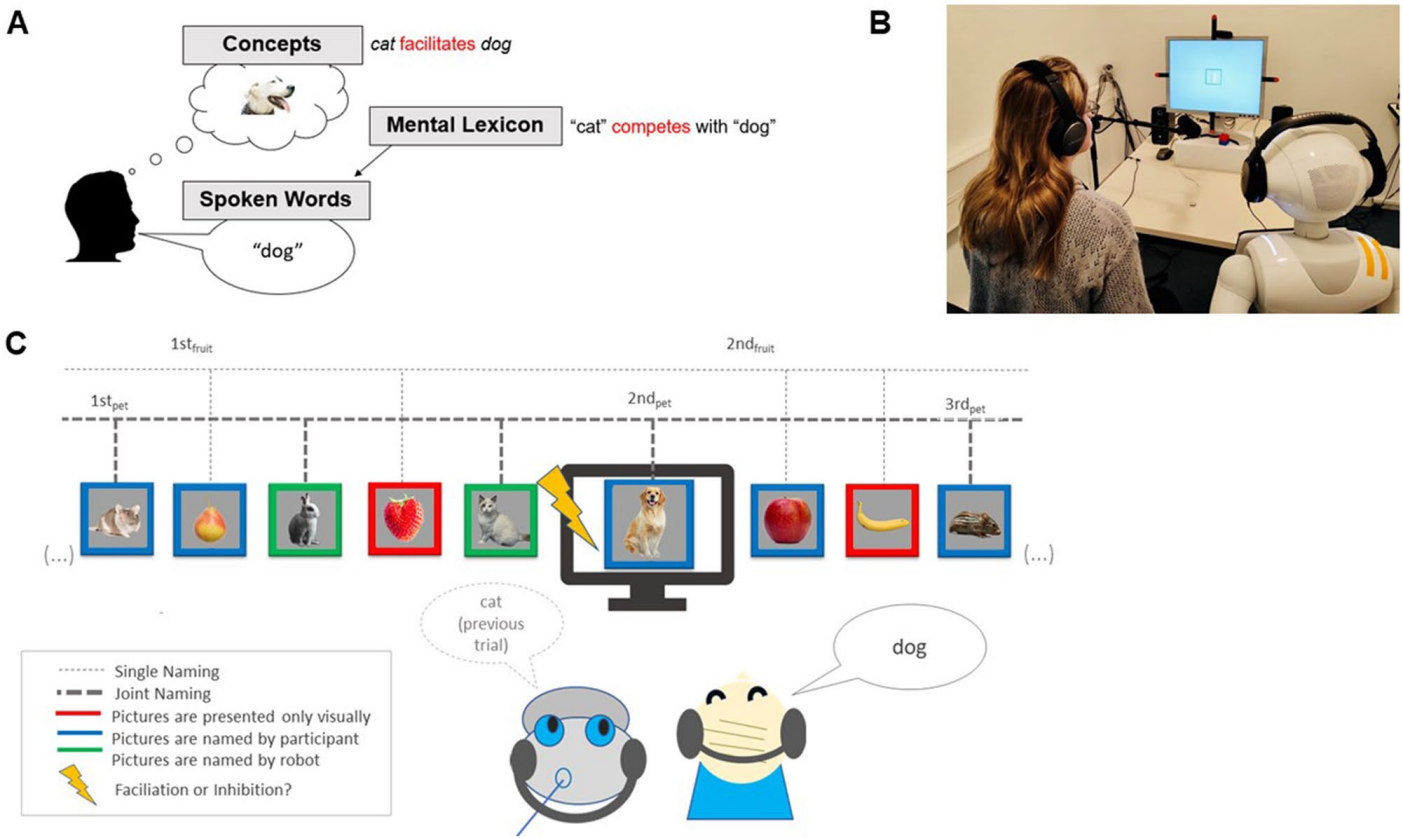
(**a**) The cognitive model of lexical access during speech production explains how concepts activate entries in the mental lexicon that are then translated into speech. Facilitation and competition can occur during speech production on different levels (conceptual vs. lexical). (**b**) We operationalized the cognitive model in our experimental setup. A participant and the robot (Pepper, Softbank Robotics) sat next to each other in front of the computer screen, displaying pictures, some of which were semantically related. We assessed whether the robot's verbal behaviour is simulated, and at which processing level (conceptual vs. lexical). To exclude a possible confound of additional linguistic input during Shared Naming with the robot, both the participants and the robot wore headphones that masked the partner’s naming. (**c**) Pictures were continuously displayed on the computer screen. In the single naming condition, half of the pictures within a given semantic category (e.g., fruits) were named by participants, the other half were presented only visually. In the joint naming condition half of the pictures within a given semantic category (e.g., pets) were named by participants, the other half by the robot partner. Ordinal positions 1–5 were counted for those pictures named by the participants.

While different theoretical alternatives have described lexical selection, they all share two assumptions: (1) speaking includes conceptualization at the message level and selection at the lexical level, and (2) conceptually related words are co-activated at the lexical level before the best-fitting words are selected (e.g., Refs.^46,48–52^). To illustrate this process, consider the course of a conversation about pets during which a speaker may activate the concept *dog* and related concepts (e.g., *cat, rabbit*), resulting in the co-activation of words representing the concepts of *dog* and other furry mammals such as *cat* and *rabbit*, among which the most appropriate word (“dog”) is then selected (see Fig. 1a). Facilitation (i.e., faster word production) emerges as a consequence of semantic priming during conceptualization (e.g., thinking about *cats* facilitates thinking about *dogs*). Yet, interference (i.e., slower word production) emerges as a consequence of competition among related and co-activated words in the mental lexicon (e.g., the word “cat” competes with the word “dog” for selection; e.g., Refs.^46,48^; for alternative views, see Refs.^51,52^) (Fig. 1a). Depending on the context and task demands, conceptual facilitation or lexical interference may dominate, resulting in overall facilitated or inhibited speaking latencies^53,54^.

In the present study, we employed the Continuous Picture Naming (CPN) Paradigm, a principal task that provides insights into these mechanisms, underlying the retrieval of words for speaking from the mental lexicon^55–58^. In this task, participants name pictures while the speeds at which they initiate speaking are closely monitored (see “Materials and methods”). While the pictures are presented in a seemingly random sequence, the critical context manipulation consists of the presentation of related objects belonging to a common semantic category, separated by a variable number of unrelated objects (see Fig. 1c). The repeated presentation of related objects should result in facilitation at the level of conceptualization due to semantic priming (e.g., Ref.^57^). However, due to competition for selection among related words at the level of the mental lexicon, this should also result in interference and therefore slower naming. For instance, if the cat has been named before and is co-activated upon naming a dog, the word “cat” strongly competes with the word “dog” for selection. This competition is increased by each additional member of the category of animals being named, resulting in an increase of naming times with each new category member named (i.e. with each Ordinal Position of the category’s member). As a consequence, the repeated naming of objects from the same semantic category results in increasingly more effortful lexical selection, a robust empirical effect referred to as cumulative semantic interference.

### Co-representation during joint language production

More recently, the CPN task has also been employed to shed light on how human communication partners are co-represented during joint picture naming^25,26^. In these studies, two participants took turns naming pictures. The results of these studies revealed that naming times slow down not only for each semantic category member named by the participant (i.e. cumulative semantic interference, as reflected by a main effect of Ordinal Position), but also, crucially, for each semantic category member named by the partner (i.e. Ordinal Position *x* Naming Condition interaction). This partner-elicited semantic interference suggests the occurrence of the simulation and co-representation of the partner’s verbal behaviour at the level of lexical selection.

Crucially, variations of this effect allow insights into the partner's co-representation at the level of conceptualization or at the level of lexical selection. As described above, semantic co-activation leads to facilitatory priming effects during conceptualization and inhibitory effects of competition during lexical selection. While the interference caused by the partner’s naming indicates that individuals co-represent their human partners by simulating their partner’s verbal behavior down to the level of lexical access^26^, the critical question that remains to be answered is: Is a robot partner’s verbal behaviour simulated in a similar way?

### Current study

To answer this question thirty-six young adults were introduced to a socially interactive humanoid robot—Pepper (Softbank Robotics)—and together with the robot completed the CPN task^26^, see Fig. 1b. The participants named a stream of pictures, embedded in which were objects of the same semantic categories. Some of these categories were named together with the robot partner, others were named solely by the participant (see Fig. 1c). In both instances half of the objects presented within each category were named by the participant and the other half were not named by the participant. Instead, these were either named by the robot partner (Shared Naming) or presented visually, but neither named by the participant nor the robot (Single Naming). The robot was physically present and stood directly next to the participants throughout the experiment. Crucially, to capture a simulation of the partner’s utterances (and not merely the representation of what the partner is saying based on the perceptual evidence) participants wore noise-canceling headphones (Fig. 1b). Thus, participants were aware that the robot was speaking but they could not hear what the robot was saying (for a similar procedure, see Ref.^26^, Experiment 3). At the end of each session, participants completed questionnaires including ratings regarding perceived robot intentionality (see “Materials and methods”, and Supplementary Information for details).

We assessed whether a robot’s verbal behaviour is co-represented in the CPN task, and how it affects language production (“Robot as a task partner experiment”). We put forward the following hypotheses (pre-registered at https://aspredicted.org/va9nr.pdf): if the robot is co-represented similar to a human task partner, we expect participants’ naming latencies to increase not only in response to the number of semantically related pictures participants previously named themselves (Single Naming), but also, additionally, in response to semantically related pictures named by the robot (i.e. as in Ref.^26^, Naming Condition × Ordinal Position Interaction during Shared Naming). The steeper increase for categories co-named with the robot would reveal that objects named by the robot elicit comparable lexical processes to naming the objects by oneself, and would thus reveal a co-representation of a robot partner down to the level of lexical selection (as in Ref.^26^). If, however, the robot’s verbal behaviour is not co-represented during the task, we expect no partner effects of the robot’s naming on the participants’ naming times.

In addition, though not previously predicted nor pre-registered by us, a main effect of Naming Condition (i.e. Single Naming vs. Shared Naming) would indicate that a robot's naming is co-represented in the task, but differently than that of a human partner. For example, if a robot’s verbal co-representation occurs at a less deep and concrete verbal level, that is, only during the stage of conceptualization but not during lexical selection, facilitation of naming responses by the robot’s co-naming of semantic categories would be the result, as during conceptualization semantic priming instead of competition dominates (Refs.^53,54^, Fig. 1a).

As previous studies emphasized perceived robot intentionality as an important factor necessary for robot co-representation (for a review see Ref.^10^), we additionally explored whether the effects are modulated by perceived robot intentionality (“Robot as a task partner experiment: intentionality”)*.* Lastly, we compared the current data where the robot acted as the naming partner with data where a human acted as the naming partner (Robot as a task partner and human as a task partner experiments). This analysis was achieved by pooling the current data with the data from Experiment 3 of Ref.^26^. This analysis was not previously pre-registered.

## Materials and methods

### Pre-registration

The experimental procedures, hypotheses, data exclusion criteria, and analyses approaches were pre-registered prior to data collection (https://aspredicted.org/va9nr.pdf).

### Participants

Thirty-six native speakers of German (26 females, 10 males) aged between 19 and 35 (mean = 25.6, SD = 4.6) were included in the data analyses. The sample size was determined via a-priori power analysis. Based on the effect sizes observed in the previous study (Ref.^26^, Experiment 3), we simulated the outcome of the anticipated LMM with 1000 iterations (R Simr package). With 36 participants, we reached a power estimate of 81.90% chance (95% confidence interval: 79.37, 84.24) for detecting an interaction between Ordinal Position and Naming Condition.

Participants with an error rate greater than 20% were excluded and replaced (n = 3). Additionally, we excluded and replaced a participant (n = 1) who, at the end of the experiment (see “Auditory input cancellation”), reported hearing a word named by the robot.

All participants gave written informed consent and were reimbursed or received course credit. The study was approved by the ethics committee of the Department of Psychology at Humboldt-Universität zu Berlin. The study was conducted in accordance with the Declaration of Helsinki (2013).

### Robot

The robot used in the experiment as the task partner was Pepper (Softbank Robotics): a 1.2 m-tall humanoid robot designed for social interaction with humans.

### Stimuli

The experimental stimulus-set of the continuous Picture Naming Paradigm (CPN) was derived from the previous study^26^. Namely, 320 photographs of natural or man-made objects mapping onto 32 different semantic categories (e.g., flowers, buildings, birds), containing 10 exemplars each, were used. In addition, 120 items, unrelated to the target categories, were used as fillers. The pictures were presented on a grey background and were 3.5 cm × 3.5 cm in size.

### Design

Stimulus lists specifying the order in which pictures were named were derived from Ref.^26^. Lists were individually created for each participant considering the following rules: (1) The order of items within one category was randomly selected (by the program ‘Mix’^59^); (2), the items of one category were randomly separated by a minimum of two and a maximum of six unrelated items (i.e., items of a different category or filler items); (3) categories belonging to the same superordinate category did not overlap within the list (e.g., birds and hoofed mammals, merging to superordinate category animals were not used in the same list).

Participants named half of the items of a given category. The other half of the items were either named by the robot (Shared Naming), or by nobody, and displayed visually only (Single Naming). The assignment of trials in which the participant, the robot, or nobody named the objects was random, with the following exceptions: the first and the last items of the same category were always named by the participant; participant’s trials were separated by maximum three trials in which either the robot or nobody named the objects. All filler items were assigned to participants’ trials.

Each participant was presented with the complete set of pictures (440 trials) twice (two experimental blocks) thus, each participant completed 880 trials. Objects assigned to participants in the first block, were the same as in the second block, however, the individual exemplars appeared in a different ordinal position.

### Auditory input cancellation

To exclude the possibility of a confound of receiving an additional linguistic (auditory) input in the Shared Naming Condition as compared to the Single Naming Condition, throughout the experiment, the participants wore noise cancelling headphones (Bose QuietComfort 25) continuously displaying pink noise (similarly to Ref.^26^, Experiment 3). To enhance the impression that the robot would also not hear the participant, the robot also wore headphones.

To not eliminate the impression that the robot is naming the words during the experiment, we aimed to have the participants hear that the robot speaks, but not have them hear the exact words that the robot is saying. To achieve this goal, the noise level was individually tested and adjusted for each participant. Before the start of the experiment, participants underwent a procedure in which the robot named seven words, one of which was an animal, and the participant had to indicate what animal was named by the robot. In case participants were able to hear an animal, the procedure was repeated with an increased volume of Pink Noise until they were no longer able to specify an animal named by the robot. The sound check was repeated once again in the middle of the experiment and 5 times at the end of the experiment. In case that participants were able to hear an animal named by the robot, in at least one of the final 5 trials at the end of the experiment, participants’ data were excluded from the analyses (n = 1) and replaced.

### Procedure

The experimental task instructions are available at: https://osf.io/qakbv/.

At the beginning of each experimental session, the robot was introduced to the participants as their task partner. The robot was introduced as a humanoid and quite communicative robot. Participants were told that the robot can recognize them and follow them with its gaze. Consistently, they were shown how: (1) the robot can follow the *experimenter* with its gaze, (2) the robot can follow the *participant* with its gaze. Furthermore, as participants sat at the chair (and were encouraged to say‚ Hello’ to the robot), the robot looked directly at the participant and said: ‘*Hello I am Pepper. I am learning German and I am learning to name different objects. We will now complete a task together*’. The emphasis on learning and object recognition, was aimed at enhancing the impression of intentionality behind robot’s behaviour, which consistently with previous research is linked to observation of social effects in experimental studies (for a review see Ref.^10^).

Following robot introduction, in a separate room, participants were given time (approximately 10 min) to familiarize themselves with the pictures that were going to be presented in the task and their target names. The pictures were presented on paper in an unsorted order.

During the experiment, participants were seated next to the robot in front of a computer screen displaying the pictures. The robot’s speech and behaviour were generated via the NAOqi framework with custom scripts that communicate between the Pepper robot and the Presentation software that visually displayed the stimuli. Pictures appeared on the computer screen, one at a time. A colored frame around the picture indicated whose turn it was to name the picture: the participant’s, the robot’s or nobody’s. The color assignment was counterbalanced across participants. Participants were instructed to name the pictures as fast and as accurately as possible in response to their ascribed color. In the remaining trials, participants were instructed not to take any action. Participants first completed a 15-trial practice session. Subsequently, participants completed two experimental blocks. Each block consisted of 440 trials, and participants were given a short break after 220 trials, and a longer break after the first block. To highlight robots’ natural behaviour, during the breaks, the robot was set to respond to and to attend to its environment. During the CPN task, the robot exhibited subtle arm and head movements (instead of being fully static during picture naming).

Each trial began with a presentation of the fixation cross for 0.5 s. In the trials in which participants named the objects, the picture was presented until the naming response was initiated or for a maximum of 2 s. Participants’ naming latency (reaction time) was recorded with the help of a voice-key from the onset of the picture presentation. In the trials in which the robot named the pictures, robot’s naming response times were variable and were modelled after average response times for a given picture recorded in^26^ and Experiment 1 of^60^. To keep the control condition (in which nobody names the pictures) comparable, the pictures in this condition were also modelled to disappear after the duration of average naming latencies for the given objects based on response times recorded in Ref.^26^ and Experiment 1 of Ref.^60^. Following each Naming Condition, a blank screen of 2 s followed each picture presentation, after which the next trial followed.

Throughout the experiment, the experimenter coded failures of the voice key as well as erroneous trials (wrong picture naming, naming at the wrong turn).

At the end of the experiment, participants completed two questions pertaining to perceived robot intentionality (derived from Ref.^19^): “The robot acted intentionally” and “The robot decided actively when to respond to a stimulus” on a scale from 1 (not at all) to 11 (extremely). Additionally, they completed a battery of questionnaires including robot perception (Goodspeed Questionnaire^61^; RoSAS^62^; HRIES^63^), attitudes towards technology questionnaire (ATAI^64^) and three questions regarding previous experience with AI, Robots and Pepper specifically (see Supplementary Information for an overview). Finally, participants were debriefed and compensated for their participation.

### Statistical analyses

#### Confirmatory analyses

Firstly, we assessed the effects of having a robot as a task partner (versus naming the pictures alone) on naming latencies to semantically related pictures.

LMMs, as implemented in the lmer function of the lme4 package for R^65^ with random effects modeled for participants and items were applied to naming latencies treated with log transformation (applied following the Box-Cox procedure^66^). Naming latencies for the trials in which the participants named the pictures were modelled as a function of the predictors: Naming Condition (Shared Naming vs. Single Naming), Ordinal Position, (ordinal position 1–5), and Experimental Block (1–2). The predictors Naming Condition and Block were contrast-coded using the sliding difference contrast. The predictor Ordinal Position was centered and entered as a continuous variable. Models were initially run with a maximum random effects structure allowed by the experimental design for participants and items^67^. Using singular value decomposition, the initial full random effect structure was simplified until the maximal informative and converging model was identified.

#### Exploratory analysis

To assess whether perceived robot intentionality affected the degree to which participants would be influenced by their robot partner’s naming response, we additionally carried out an LMM adding the perceived robot intentionality score as a predictor to the original model. As in Ref.^19^, perceived intentionality score was calculated by averaging the responses to the questions: “The robot acted intentionally” and “The robot decided actively when to respond to a stimulus”, as the two items appeared to measure the same construct (Cronbach’s alpha = 0.89). The predictor Intentionality (1–11) was subsequently centered and added to the original model as a continuous variable.

#### Additional analysis: comparing robot and human partner

To compare the effects of naming the semantic categories together with a robot partner to naming the semantically related objects together with a human partner, we pooled the current data with the data obtained from Experiment 3 in Ref.^26^. In this experiment with human task partners, 36 participants between the ages of 18 and 36 (mean 25.14 years; 9 males, 27 females) participated^26^. The two experiments differed in the following aspects: the experiment with human partners^26^ consisted of 1 block only (i.e., 440 trials). Thus, to make the two datasets comparable, for this analysis, we only included the data from block 1 of the current experiment (i.e., 440 trials). While the timing of the disappearance of the objects in the solo condition in the current experiment was modelled after naming responses observed in the previous studies utilizing the paradigm (Ref.^26^, Experiment 1^60^), the timing of disappearance in the experiment with human partners (Experiment 3^26^) reflected average reaction times recorded for Experiment 1 of the same study. The efficacy of the headphones was assessed 3 times in the current experiment (prior to experiment, after block 1, after block 2, whereas in the experiment with human partners, it was assessed only prior to the experiment. While in the experiment with human partners, participants who reported understanding the words named by the partner in the experiment were excluded from the analysis, in the current experiment we used an improved procedure to assess whether the participants were able to hear the words named by the robot to exclude participants based on auditory input (see “Auditory input cancellation”).

For LMM analysis, the predictor Experiment was contrast-coded using the sliding difference contrast and added to the maximal LMM model. Using singular value decomposition, the initial full random effect structure was simplified until the maximal informative and converging model was identified. This analysis was not previously pre-registered.

## Results

### Robot as a task partner experiment

**Figure 2:**
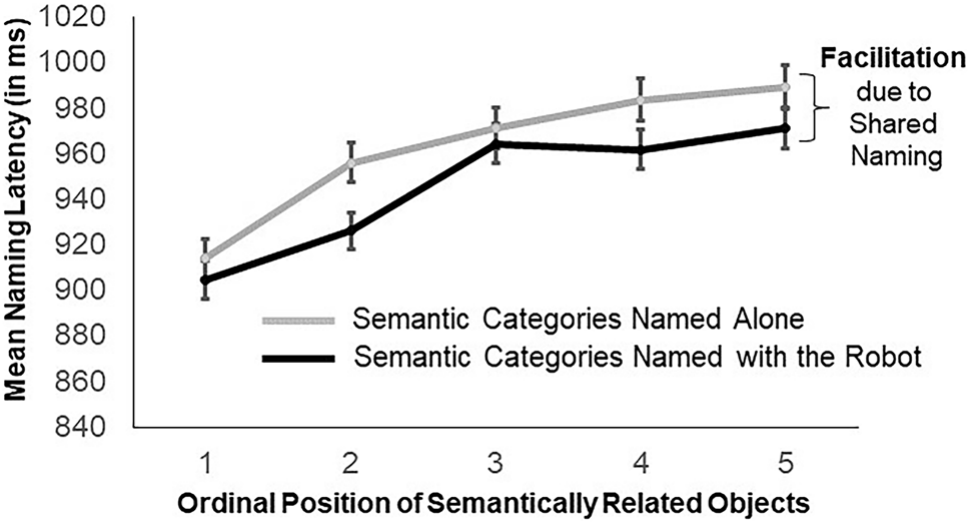
Naming latencies in the naming task with a robot partner. Mean naming latency and standard errors (in milliseconds) observed in the current experiment with a robot partner, broken down by ordinal position and naming condition. Faster naming latencies for semantic categories named with the robot (Shared Naming) as compared to semantic categories named alone (Single Naming) point to a facilitatory effect of sharing the naming task with the robot on speaking.

Participants’ naming latencies in the CPN task increased with each ordinal position within semantically related categories by an average of 18 ms. A main effect of Ordinal Position confirmed the cumulative semantic interference effect, demonstrating that the paradigm produces the well-known effect also in the general presence of a robot partner. Crucially, we observed a 17.2 ms facilitation for semantic categories co-named with the robot (Shared Naming) as compared to the categories named alone (Single Naming). Specifically, naming latencies were faster for those semantic categories that were also named by the robot (M = 945.8, SD = 282.1) than for those semantic categories that were only named by the participant (M = 963.0, SD = 293.4, Fig. 2). A main effect of Naming Condition confirmed the influence of having a robot as a task partner on facilitated naming. Lastly, participants named the pictures faster in block 2 (M = 915.80, SD = 360.83) as compared to block 1 (M = 994.10, SD = 406.62), as revealed by a significant main effect of Block. For an overview of Linear Mixed-effects Models (LMM) effects see Table 1.

**Table 1:** LMM for the robot as a task partner experiment.

	Est/Beta	SE	df	t	p
**Fixed effects**					
Intercept	6.83	0.02	55.94	368.70	< 0.001
Naming condition	0.02	0.00	296.9	3.60	< 0.001
Ordinal position	0.02	0.00	10,080	11.61	< 0.001
Block	− 0.08	0.00	10,030	− 19.19	< 0.001
Naming condition × ordinal position	0.00	0.00	10,260	− 0.48	0.632
Naming condition × block	0.01	0.01	10,120	0.76	0.447
Ordinal position × block	− 0.01	0.00	10,120	− 1.61	0.108
Naming condition × ordinal position × block	0.00	0.01	9806	0.50	0.614

	Variance	Std. Dev			
**Random effects**					
**Item**					
Intercept	0.02	0.15			
Naming condition	0.00	0.01			
**Subject**					
Intercept	0.01	0.10			
Residual	0.05	0.23			
**Model fit**					
Log likelihood	251.3				
Deviance	− 502.7				
Model equation: log(naming latencies) ~ naming condition × ordinal position × block + (1 | subject) + (naming condition | item)					

Fixed-effect estimates, standard errors, degrees of freedom, t-values and p-values for the selected model of the Robot as a Task Partner Experiment’s analyses, as well as estimates of the variance and square root (standard deviations) of the random effect structure and goodness-of-fit statistics.

### Robot as a task partner experiment: intentionality

Participants’ mean response to the question “The robot decided actively when to respond to a stimulus” was 6.6 (SD = 3.5, range 1–11, 11 = very intentional) and to the question “The robot acted intentionally” it was 6.6 (SD = 3.3, range 1–11, 11 = very intentional). Adding the average score of perceived robot intentionality to the LMM model did not result in a significant modulation of the effects.

### Comparing robot as a task partner and human as a task partner

**Figure 3:**
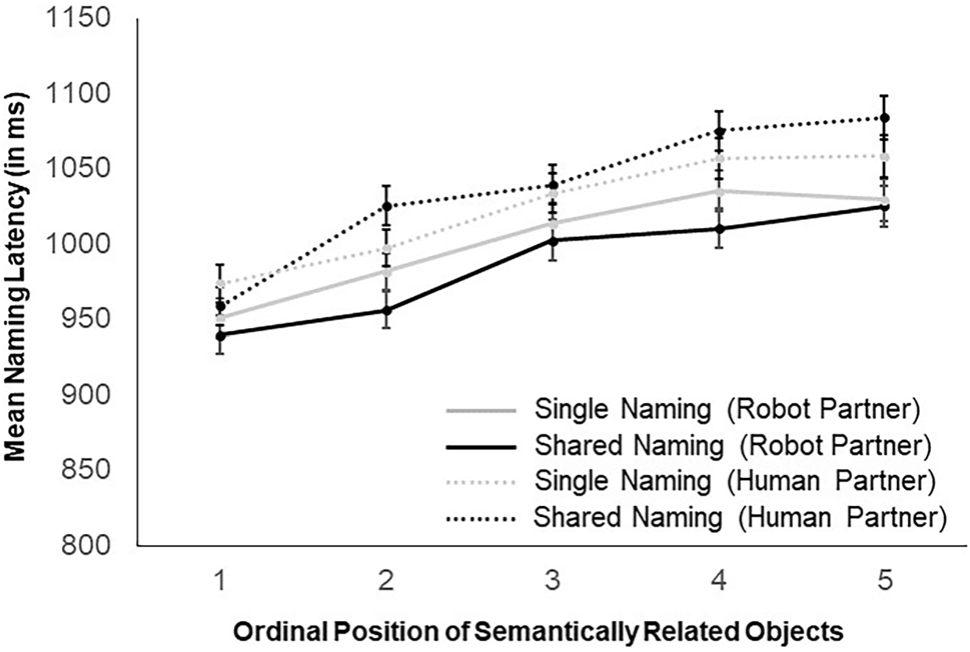
Naming latencies in the naming tasks with a robot and a human partner. Mean naming latency and standard errors (in milliseconds) observed in the current experiment with a robot partner (Block 1) and in experiment 3 from Ref.^26^ with a human partner, broken down by ordinal position and naming condition.

Across the pooled data set including the current experiment with a robot task partner and an experiment with a human task partner^26^, naming latencies increased with each ordinal position within semantically related categories by an average of 23 ms (see Fig. 3). The main effect of Ordinal Position confirmed the cumulative semantic interference effect in the two experiments. In addition, naming latencies were faster, in general, in the experiment in which the task partner was a robot (M = 994.4, SD = 299.7, Block 1) as compared to the experiment in which the task partner was a human (M = 1030.1, SD = 293.8), as revealed by a main effect of the Experiment. Crucially, as revealed by a Naming Condition by Experiment interaction, participants were faster to name semantically related pictures with a robot partner (M = 986.7, SD = 294.2) compared to a human partner (M = 1036.4, SD = 296.83), while naming semantic categories alone was more comparable in the robot (M = 1002.1, M = 305.24) and the human (M = 1023.8, M = 290.9) experiments (see Fig. 3). The LMM analyses confirmed these effects (see Table 2 for a summary).

**Table 2:** LMM for the combined robot and human datasets.

	Est/Beta	SE	df	t	p
**Fixed effects**					
Intercept	6.89	0.02	65.36	432.54	< 0.001
Naming condition	0.00	0.00	9812	0.350	0.7262
Ordinal position	0.02	0.00	9899	13.488	< 0.001
Experiment	0.05	0.00	9845	9.543	< 0.001
Naming condition × ordinal position	0.00	0.00	9819	− 1.207	0.2275
Naming condition × experiment	− 0.02	0.01	9812	− 2.517	0.0119
Ordinal position × experiment	0.00	0.00	9827	1.463	0.1435
Naming condition × ordinal position × experiment	0.00	0.01	9820	− 0.550	0.5826

	Variance	Std. Dev			
**Random effects**					
Item (intercept)	0.02	0.15			
Subject (intercept)	0.01	0.08			
Residual	0.06	0.23			
**Model fit**					
Log likelihood	− 261.0				
Deviance	522.0				
Model equation: log (naming latencies) ~ naming condition × ordinal position × experiment + (1 | subject) + (1 | item)					

Fixed-effect estimates, standard errors, degrees of freedom, t- values and p-values for the selected model of the Robot as a Task Partner and Human as a Task Partner Experiments; estimates of the variance and square root (standard deviations) of the random effect structure and goodness-of-fit statistics are also presented.

## Discussion

The aim of this study was to assess whether humans co-represent a social robot’s verbal behaviour in a joint verbal communication task, and to identify the consequences of robot verbal co-representation on language production. To this end, human participants named semantically related pictures together with a robot task partner. Half of the semantic categories underlying these pictures were named by the participant only, the other half was named together with the robot partner. We found facilitatory effects of sharing the naming task with the robot on language production, pointing to: (1) co-representation of the robot partner during a shared verbal task, and (2) facilitatory effects of robot verbal co-representation at the level of conceptualization, where the meaning of the intended verbal message is generated. Crucially, this is not a mere presence effect, but an effect specific to sharing a task with a robot partner, only seen for semantic categories that were co-named together with the robot.

Contrary to previous findings with human partners^25,26^, we did not observe enhanced cumulative interference due to shared naming together with the robot partner. Instead, naming semantic categories together with the robot led to facilitation, as revealed by faster naming of semantic category members co-named with the robot. This difference was corroborated in analyses directly comparing the data from experiments in which the task partner was a robot and in which the task partner was a human: while naming was facilitated with a robot partner, naming was not facilitated by human partners, thus revealing the specificity of the facilitatory effect to sharing the task with a robot partner.

These findings demonstrate that a robot`s verbal behaviour is co-represented, yet differently from that of humans. As introduced above, language production models distinguish between the meaning-based level of conceptualization and the lexical level of word selection (e.g., Refs.^47–52^). The co-activation of concepts may facilitate the generation of the message, whereas, in parallel, the co-activation of alternative words may inhibit the selection of the best-fitting word due to competition from related alternative words. The observation of a facilitation effect induced by a robot task partner suggests that robots are not co-represented down to the level of lexical selection (as humans do^22,26^). Instead, robot's language appears to be simulated only at the conceptual level, resulting in a facilitatory effect on language production due to priming. This more `shallow’ co-representation is also in line with previous findings on linguistic alignment showing that lexical entrainment happens for both human and robot partners, yet that the robot’s influence is weaker than that of the human partner^38^. Since the Continuous Picture Naming task has been employed with human and robot partners, we conclude that robots are co-represented, but the simulation seems to include only conceptualization on their behalf, and not lexical selection. This meaning-based simulation of the robot partner helps speakers to generate their own verbal message, which in turn facilitates speaking.

A note should be made that a related line of research^24^ claimed that speakers do not co-represent the content of their partners’ utterances, but instead they represent merely the act of each other’s naming. We complement this line of research by demonstrating that the representation of conceptual and/or lexical content of the partner’s task can be observed. Just as the baseline effects of semantic effects in this task, it is the previous experience of having named a related object or the experience of simulating conceptual or lexical processing on behalf of the task partner that elicits these effects in the present task. Thus, in comparison to the study by Ref.^24^, the current paradigm includes (implicit) memory of processing objects either directly or on behalf of the task partner. We have built our predictions on the above-mentioned language production models specifically assuming interference via accessing semantically related items at the level of the mental lexicon and facilitation at the level of concepts (e.g. Refs.^53,54^). Consistently, the observed effects (i.e. partner-elicited inhibitory effects with a human partner^26^; and facilitatory effects with a robot partner, observed here) are in line with these predictions, suggesting co-representation of the *content* of the partner’s speech. The exact nature of this co-representation is still nonetheless unknown and should be addressed in future studies by, for example, employing physiological measures, such as EEG, specifically targeting the underlying mechanisms (also see Ref.^60^).

Future research should also reveal further insights into the critical differences and potential commonalities between human and robot verbal co-representation, utilizing between or within-subjects designs. Since in the current study we did not find a modulatory influence of perceived robot intentionality on robot verbal co-representation, this factor does not seem to play a major role. Future studies shall also systematically investigate whether the specifics of a robot’s behavior, (synthetic) voice or visual appearance, as well as the intensity of verbal interactions or enhanced knowledge about the robot’s attributes may be relevant factors affecting robot verbal co-representation. Although in the current study we performed repeated checks to assure that participants could not hear the exact words named by the robot (and thus that the effects were driven by a simulation of robot’s speech rather than linguistic processes), future studies should consider having artificial agents produce phonologically acceptable non-words instead of having them name real words during the experiment.

To conclude, not only can the verbal behaviour of robots be simulated during HRI, sharing a verbal task with a robot also facilitates speaking. These results offer important insights into contexts in which verbal communication with robots is prevalent. The findings might also be of special importance for clinical populations where speech delays and lexical retrieval difficulties prevail, and in educational contexts where language production enhancement is particularly desired: verbal interactions with robots in these contexts might be particularly advantageous in facilitating spoken language production.

Representation of robot partners by humans has previously been confirmed across information processing levels which include: motor (for a review, see Ref.^10^), social (for a review, see Ref.^68^), and higher-order cognitive levels^69^. Our results provide a crucial extension to the increasingly relevant domain of robot verbal co-representation by providing insights into the nature of how robots are simulated in shared tasks, and how it affects human behavior. This advances our path towards characterizing the intricacies of real-world human–robot social interactions, which currently remains one of the grand challenges of the field of robotics^1,70^. The mechanisms of co-representation and language facilitation revealed here might be integrated into a cognitive architecture for social robots to enhance their communicative skills in verbal interactions with humans^10^.

## Supplementary Information

Supplementary Information.

## Data Availability

The datasets generated and analyzed during the current study are available in the Open Science Framework repository: https://osf.io/qakbv/.
