# Supplementary material for: Robots facilitate human language production
Source: Sci Rep. 2021 Aug 18;11:16737. doi: 10.1038/s41598-021-95645-9 (PMC8374029; doi:10.1038/s41598-021-95645-9)
Supplement: Supplementary file 1 — Supplementary Information. [file 41598_2021_95645_MOESM1_ESM.pdf]

Supplementary Materials for

Robots facilitate human language production

Olga A. Wudarczyk*, Murat Kirtay, Doris Pischedda, Verena V. Hafner, John-Dylan Haynes, Anna K. Kuhlen #, Rasha Abdel Rahman#

#Kuhlen and Abdel Rahman should be considered joint senior author

*Correspondence to: wudarczo@hu-berlin.de

Supplementary Table: Participants' attitudes towards technology, robot perception and experience with robots.

	**Mean (SD)**	**Range of the scale (1=low)**
**ATAI**		
**Acceptance**	7.2 (1.9)	1-11
**Fear**	4.3 (1.8)	1-11
**GODSPEED**		
**Anthropomorphism**	11.7 (4.4)	1-25
**Animacy**	17.8 (4.4)	1-30
**Likeability**	20.8 (3.2)	1-25
**Perceived Intelligence**	18.0 (4.0)	1-25
**Perceived Safety**	11.0 (1.7)	1-15
**ROSAS**		
**Discomfort**	2.6 (1.6)	1-9
**Warmth**	3.4 (1.7)	1-9
**Competence**	6.1 (1.6)	1-9
**HRIES**		
**Sociability**	4.4 (1.4)	1-7
**Animacy**	2.9 (1.3)	1-7
**Agency**	4.2 (1.3)	1-7
**Disturbance**	2.0 (0.9)	1-7
**Previous Experience with…**		
**With Pepper Robot**	1.7 (1.9)	1-11
**With Robots**	2.6 (2.1)	1-11
**With AI**	4.0 (2.6)	1-11
**Intentionality**		
**"The robot decided actively when to respond to a stimulus"**	6.6 (3.5)	1-11
**"The robot acted intentionally"**	6.6 (3.3)	1-11
**Intentionality (overall):**	6.6 (3.2)	1-11

Table caption: Mean scores and standard deviations (in parentheses) for the Participants' attitudes towards technology (ATAI, [64]), robot perception (Godspeed, [61]), RoSAS [62], and HRIES [63] questionnaires); previous experience with robots and perceived robot intentionality (based on [19]).
